# College and Hospital Notes

**Published:** 1903-03

**Authors:** 


### COLLEGE AND HOSPITAL NOTES.

THE laboratory plans for the Rockefeller Institute for Medical Research have been announced. It is to be located in New York and the scheme contemplates a single laboratory building, arranged to permit of such extension as the growth of the work may demand. Professor Simon Flexner, of Philadelphia, will have charge of the work as director, and there will be directors of the different departments—namely, physiological chemistry, hygiene and preventive medicine, pharmacology and therapeutics, normal and pathological physiology, and bacteriology. The work on the laboratory building will commence at once.

In order to ally the research work of the institute closely with practical medicine, it is the purpose of the directors to erect in the near future a hospital where special groups of patients may be treated, as it is one of the functions of the institute not only to discover the causes of disease, but to develop new methods of treatment. This hospital will not be large, but will be fully equipped, so that the best possible care can be given to the special cases under treatment. Plans for this new building are already under consideration, and it is expected that the first laboratory will be ready for work by October, 1904.

THE Buffalo General Hospital has received recently two generous gifts. Mr. Frank H. Goodyear has donated $50,000 and Mr. William Hamlin has sent a check of $49,900. The trustees have raised $25,750 among themselves and the public is asked to subscribe $74,350, in order to make a total of $200,000, the amount which the hospital trustees say they need.

In an address to the public the trustees say there is a mortgage for $50,000, a floating debt of $88,000, necessary improvements to buildings will cost $12,000, and $40,000 is needed for a hospital for infectious diseases. Subscriptions, large or small, should be sent to Charles R. Wilson, treasurer, 4 White Building, Buffalo.

